# Cyclin-Dependent Kinase Regulatory Subunit 2 Indicated Poor Prognosis and Facilitated Aggressive Phenotype of Hepatocellular Carcinoma

**DOI:** 10.1155/2019/8964015

**Published:** 2019-10-22

**Authors:** Jie Zhang, Qianqian Song, Jinxia Liu, Lina Lu, Yuqing Xu, Wenjie Zheng

**Affiliations:** ^1^Department of Chemotherapy, Affiliated Hospital of Nantong University, 20 Xisi Road, 226001 Nantong, Jiangsu, China; ^2^Research Center of Clinical Medicine, Affiliated Hospital of Nantong University, 20 Xisi Road, 226001 Nantong, Jiangsu, China; ^3^Department of Radiology, Wake Forest School of Medicine, One Medical Center Boulevard, Winston-Salem, 27157 NC, USA; ^4^The Key Laboratory of Systems Biology, CAS Center for Excellence in Molecular Cell Science, Shanghai Institute of Biochemistry and Cell Biology, Chinese Academy of Sciences, University of Chinese Academy of Sciences, 200031 Shanghai, China

## Abstract

Cyclin-dependent kinase regulatory subunit 2 (CKS2) is a member of the cell cycle-dependent protein kinase subunit family, which is implicated as an oncogene in various malignancies. However, the clinical significance, oncogenic functions, and related mechanisms of CKS2 in hepatocellular carcinoma (HCC) remain largely unclear. In the present study, expression features and prognostic value of CKS2 were evaluated in the bioinformatic databases and HCC tissues. The effects of CKS2 on the malignant phenotypes of HCC cells were explored *in vitro*. According to the analyses of three bioinformatic databases, mRNA levels of CKS2 were elevated in HCC tissues compared with the normal tissues. Immunohistochemical assays found that high CKS2 expression was closely associated with liver cirrhosis (*P* = 0.019), poor differentiation (*P* = 0.02), portal vein invasion (*P* < 0.001), TNM stage (*P* = 0.019), tumor metastasis (*P* = 0.008), and recurrence (*P* = 0.003). The multivariate regression analyses suggested that CKS2 was an independent prognostic factor for overall survival (HR = 2.088, *P* = 0.014) and disease-free survival (HR = 2.511, *P* = 0.002) of HCC patients. Moreover, the bioinformatic analyses indicated that CKS2 might be associated with the malignant phenotypes in HCC progression. In addition, *in vitro* assays showed that CKS2 expression was higher in HCC cell lines than in normal liver cells. Knockdown of CKS2 remarkably repressed the proliferation, colony formation (*P* = 0.0003), chemoresistance, migration (*P* = 0.0047), and invasion (*P* = 0.0012) of HCC cells. Taken together, overexpression of CKS2 was significantly correlated with poor prognosis of HCC patients and the malignant phenotypes of HCC cells, suggesting that it was a novel prognostic biomarker and potential target of HCC.

## 1. Introduction

Hepatocellular carcinoma (HCC), accounting for 85–90% of all primary liver cancers, is the sixth most common type of cancer as well as the third most frequent cause of cancer-related deaths [1, 2]. Due to the infection of hepatitis B virus (HBV) or hepatitis C virus (HCV), HCC occurs more frequently in developing countries compared with developed countries [3]. Liver transplantation and radiofrequency (thermal) ablation (RF(T)A) are commonly applied in HCC patients at early and intermediate stages [4–6]. Despite the great efforts on pathology and physiology of HCC, it remains unclear for the molecular mechanisms underlying aggressive behaviors of HCC. Sorafenib, a multiple tyrosine kinase inhibitor, is the only systemic agent approved by the FDA for the first-line treatment of unresectable HCC patients [7]. While various targeted drugs (regorafenib, lenvatinib, and nivolumab) have been adopted in the treatment paradigm, the long-term survival of patients with HCC remains poor [8–10]. Therefore, it is of great importance to find novel prognostic biomarkers and a potential target for HCC.

Cdc kinase subunit (CKS) proteins are small (9 kDa) highly conserved cyclin-dependent kinase (CDK) binding proteins, which are essential components for cell cycle regulation [11, 12]. The CKS family consists of two members, CKS1 and CKS2. CKS1, a well-known cell cycle-related protein, has been implicated in various tumors, including breast, lung, liver, and prostate cancers [13–16]. In addition, CKS2 is also observed in the transition of the cell cycle in multiple biological activities. Specifically, CKS2 could promote the early embryonic development and the somatic cell division [17]. However, accumulating evidence indicated that CKS2 might contribute to tumor progression [18]. Overexpression of CKS2 is determined in several cancer types and indicated a high risk of metastasis and recurrence. Though a recent study suggested the positive roles of CKS2 in biological behaviors of HCC cells [19], the potential clinical value and underlying functions of CKS2 remained largely unexplored. Based on the clinical samples and *in vitro* investigations, this study proposed CKS2 as a promising prognostic biomarker and therapeutic target for HCC.

## 2. Materials and Methods

### 2.1. Patient Information

HCC tissue samples and self-matched adjacent nontumor tissues were obtained from 156 HCC patients (19 females and 137 males; age range, 35-74 years; mean age, 50.27) who underwent hepatectomy at the Affiliated Hospital of Nantong University (Jiangsu, China) between 2008 and 2012. Of them, 133 patients (85.3%) were diagnosed as HBsAg positive, 118 patients (75.6%) with liver cirrhosis, and 54 cases (34.6%) with an advanced stage (III/IV). The stages of all the enrolled patients were classified according to the 8th tumor node metastasis (TNM) classification system of the International Union Against Cancer. None of the patients received radiotherapy or preoperative chemotherapy before surgery. All patients were followed up until December 2017. The diagnosis of HCC was confirmed histologically. This study was approved by the Ethic Committees of the Affiliated Hospital of Nantong University.

### 2.2. Data Processing

RNA-seq data for HCC was obtained from bioinformatic databases, including The Cancer Genome Atlas (TCGA, http://gdc.cancer.gov/); Gene Expression Omnibus (GEO) datasets GSE14520, GSE45436, GSE36376, and GSE54238 (http://www.ncbi.nlm.nih.gov/geo); and Oncomine databases (https://www.oncomine.org/). CKS2 mRNA-seq data was log2 normalized and analyzed by using R software. The prognostic and correlation analyses of CKS2 was obtained from the Gene Expression Profiling Interactive Analysis (GEPIA) online database (http://gepia.cancer-pku.cn/). The potential roles and interactions of CKS2 were predicted by the Cancer Hallmarks Analytics Tool (http://chat.lionproject.net/) and protein interaction analytic tool (https://genemania.org/), respectively.

### 2.3. Gene Set Enrichment Analysis

RNA-seq of HCC samples from TCGA data was divided into two groups according to the median values of the expression of CKS2 (high vs. low expression). GSEA 3.0 software (Broad Institute, Cambridge, MA, USA; http://www.broad.mit.edu/gsea) was performed with reference from the Molecular Signatures Database (MSigDB, http://software.broadinstitute.org/gsea/msigdb). Thresholds were set following permutation tests for 1000 times. *P* value and normalized enrichment score (NES) were used to sort the possible pathways enriched in each group. Functional enrichment in the KEGG pathways and GO biological processes was assessed by hypergeometric test, which was used to identify a priori-defined gene sets that showed statistically significant differences between two groups. The test was performed by the R package clusterProfiler [20].

### 2.4. Immunohistochemical Staining

HCC and self-matched paracancerous tissues from 156 cases were fixed in formalin and embedded in paraffin for immunohistochemistry (IHC). In brief, following deparaffinization with xylene and rehydration with gradient ethanol, antigen retrieval was conducted by using sodium citrate buffer solution in a microwave. After that, sections were incubated with 3% hydrogen peroxidase to block endogenous peroxidase activity. Then, sections were incubated with primary anti-CKS2 rabbit monoclonal antibody (1 : 50, Abcam, USA) overnight at 4°C. After washing with phosphate buffer saline (PBS), samples were incubated with secondary HRP antibody (Dako, Denmark) for 2 h at room temperature. Following visualization by diaminobenzidine tetrachloride (DAB) and counterstaining by haematoxylin, sections were covered by coverslips with mounting media (Dako, Denmark).

### 2.5. Immunostaining Scores

Sections were independently assessed by two experienced histopathologists. Once it came to a conflicting result, the cases were evaluated again by a third histopathologist. The scores were semiquantitatively evaluated via two scoring parts: staining intensity and positive cell ratio. The immunostaining intensity was scored as follows: 0, no staining; 1, slightly yellow; 2, yellow brown; and 3, brown. The positive cell score was determined as follows: 0 (<5%), 1 (5~40%), 2 (40~75%), and 3 (>75%). The final score was calculated as the staining intensity score plus positive cell score. A score of 0-2 was considered negative, and a score of 3-6 was positive.

### 2.6. Cell Culture and Transfection

Hep3B, SMMC7721, Huh7, HepG2, SMMC7721, MHCC97H, and LO2 were obtained from the Type Culture Collection of the Chinese Academy of Sciences (Shanghai, China). Cells were cultured in Roswell Park Memorial Institute-1640 (RPMI-1640, Gibco, USA) or Dulbecco's modified Eagle medium (DMEM, Gibco, USA) with 10% fetal bovine serum (FBS, Gibco, USA) and penicillin/streptomycin in a CO_2_ incubator. For the transfection, cells were cultured in the 6-well plate and prewashed twice with 2 ml shRNA Transfection Medium (Santa Cruz, USA). KD-CKS2 Plasmid DNA solution (Santa Cruz, USA) was gently mixed with shRNA Plasmid Transfection Reagent (Santa Cruz, USA). Then, the suspension was added into indicated wells and incubated for 8 h, followed by incubation in the complete medium for 24 h. Finally, the transfection efficacy was verified by western blotting and RT-qPCR.

### 2.7. Cell Counting Kit-8 Assay

The proliferation and viability of HCC cells was conducted using Cell Counting Kit-8 (CCK-8, Sigma, USA) according to the manufacturer's instructions. For the detection of proliferation, 1000 cells transfected with NC or KD-CKS2 were seeded in the 96-well plates. Then, the wells were exposed to CCK-8 solution at indicated time points and detected at 450 nm by using a plate reader (MD, USA). For the viability assay, the stock solution of sorafenib or regorafenib (Selleck, USA) was diluted in dimethyl sulfoxide (DMSO, Sigma, USA). Cells were treated with sorafenib at indicated concentrations for 48 h. Control group was treated with DMSO. Then, the plates were incubated with CCK-8 solution for 2 h at 37°C, followed by reading at 450 nm by a plate reader (MD, USA).

### 2.8. Colony Formation Assay

MHCC97H cells transfected with KD-CKS2 and NC were seeded in six-well plates at a density of 500 cells/well. The cells were cultured for 12 days, followed by washing in PBS, fixing in 4% paraformaldehyde, and staining in 0.5% gentian violet. Then, the colony number was calculated and presented as mean ± SD of three independent experiments.

### 2.9. Transwell Assay

MHCC97H cells transfected with KD-CKS2 and NC were harvested and resuspended in serum-free medium. For the invasion assay, 8 *μ*m Transwell chambers (Corning, USA) were precoated with Matrigel (BD Biosciences, USA) at the ratio of 1 : 5. For the migration assay, the chambers should not be pretreated with Matrigel. Then, the cells (1 × 10^5^ cells/mL) were plated in the upper chambers, while the lower chamber was added with 600 *μ*L DMEM medium supplemented with 10% FBS. Following incubation at 37°C for 24 h, cells were rinsed in PBS, fixed in 4% paraformaldehyde, and stained in 0.5% gentian violet. The cell migration or invasion was visualized and counted in 3 random fields under a microscope (Leica, USA).

### 2.10. RNA Isolation and qRT-PCR

Total RNA was isolated according to the manufacturer's instructions of RNeasy Plus Mini Kit (QIAGEN, Germany). Complementary DNA (cDNA) was synthesized by using the RevertAid™ First Strand cDNA Synthesis Kit (MBI Fermentas, CA). Real-time quantitative PCR (RT-qPCR) was performed to analyze cDNA using SYBR® Premix Ex Taq™ II (TaKaRa, Japan) according to the manufacturer's instructions, with glyceraldehyde-3-phosphate dehydrogenase (GAPDH) as a reference gene. The mRNA value was calculated using the 2^−*ΔΔ*Ct^ method. The sequences of the primers were as follows. CKS2, F: 5′-TTCGACGAACACTACGAGTACC-3′, R: 5′-GGACACCAAGTCTCCTCCAC-3′; GAPDH, F: 5′-GGAGCGAGATCCCTCCAAAAT-3′, R: 5′-GGCTGTTGTCATACTTCTCATGG-3′.

### 2.11. Western Blotting

Equal amounts of protein (30 *μ*g) were loaded in 10% sodium dodecyl sulfate-polyacrylamide gel electrophoresis (SDS-PAGE), followed by transferring to the polyvinylidene difluoride (PVDF) membranes (Millipore, USA). Then, the membranes were blocked in 5% bovine serum albumin (BSA, Sigma, USA) for 3 h and incubated in the anti-CKS2 solution (1 : 500, Abcam, USA) overnight at 4°C. Then, the membranes were washed in TBST and incubated in the IgG horseradish peroxidase conjugate secondary antibody (1 : 1000, Thermo Scientific, USA). The membranes were visualized by using the enhanced chemiluminescence (ECL) kit (Millipore, USA).

### 2.12. Statistical Analysis

The data in this study are presented as mean ± SD. These analyses were performed with SPSS 20.0 and GraphPad 7.0 software. The *χ*^2^ test was used to analyze the correlations between CKS2 expression and various clinicopathological features. Cox regression and Kaplan-Meier methods were used to analyze overall and disease-free survival of HCC patients. *P* < 0.05 was considered statistically significant.

## 3. Results

### 3.1. CKS2 mRNA Was Upregulated in Human HCC Tissues

**Figure 1:**
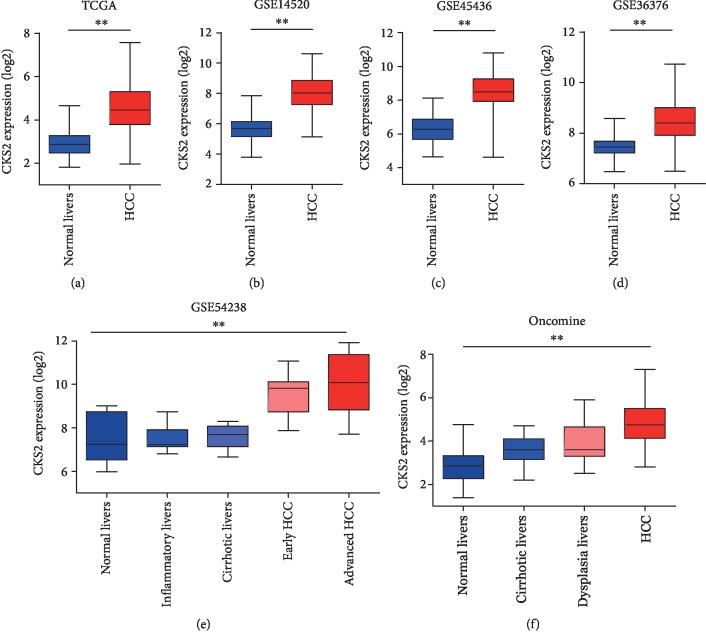
Upregulated CKS2 mRNA in HCC tissues. CKS2 mRNA levels in HCC tissues, normal liver tissues, or tissues with chronic liver diseases were extracted from several bioinformatic databases, including TCGA (a), GSE14520 (b), GSE45436 (c), GSE36376 (d), GSE54238 (e), and Oncomine (f). All data were normalized with log2. HCC: hepatocellular carcinoma; CKS2: cyclin-dependent kinase regulatory subunit 2. ^∗∗^*P* < 0.01.

CKS2 mRNA levels in HCC tissues extracted from several bioinformatic databases are shown in Figure 1. According to the TCGA database, CKS2 mRNA in HCC tissues (371 cases) were significantly higher (Figure 1(a); fold change, 3.14; *P* < 0.001) than that in normal liver tissues (50 cases). Similarly, higher CKS2 mRNA levels were also observed in HCC tissues compared with normal tissues in databases including the GSE14520 (Figure 1(b); fold change, 5.02; *P* < 0.001), GSE45436 (Figure 1(c); fold change, 4.36; *P* < 0.001), and GSE36376 (Figure 1(d); fold change, 2.07; *P* < 0.001). In addition, the CKS2 expression was significantly elevated in HCC tissues at the advanced stage compared to cases at the early stage (Figure 1(e), *P* < 0.001). Consistently, as shown in Figure 1(f), Oncomine databases demonstrated that the CKS2 expression gradually increased from normal livers (fold change, 4.12; *P* < 0.001), cirrhotic livers (fold change, 2.50; *P* < 0.001), and dysplasia livers (fold change, 1.94; *P* < 0.001) to HCC. Thus, these results indicated that CKS2 was highly expressed in HCC tissues and might be involved in the progression of HCC.

### 3.2. Immunochemical Analysis of CKS2 in HCC Tissues

**Figure 2:**
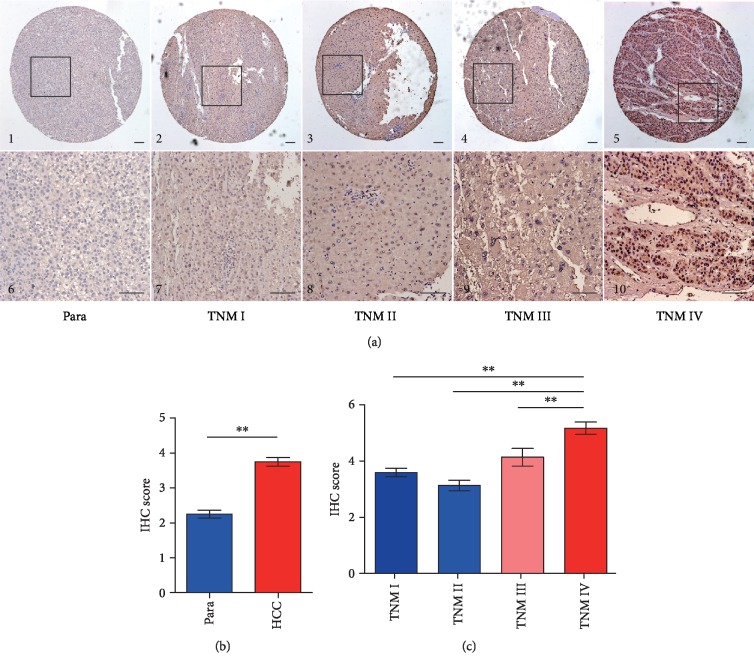
CKS2 expression in HCC tissues and paracancerous tissues by immunohistochemistry. (a) Representative immunohistochemical staining images of paracancerous tissues and HCC tissues in 156 HCC cases at different TNM stages. (b, c) Semiquantitative analysis was conducted to assess the CKS2 protein levels between HCC and paracancerous tissues or among HCC cases at different stages. HCC: hepatocellular carcinoma; para: paracancerous tissues; CKS2: cyclin-dependent kinase regulatory subunit 2; TNM: tumor node metastasis. ^∗∗^*P* < 0.01.

Immunochemical staining of CKS2 in 156 HCC and paracancerous tissues is presented in Figure 2. CKS2 was mainly distributed in the cytoplasm of paracancerous tissues, while it presented obvious nucleus staining in HCC tissues (Figure 2(a)). According to the semiquantitative analysis, higher staining intensity was determined in HCC tissues at advanced stages. However, weaker staining was observed in the paracancerous tissues or the cases at the early stage (Figures 2(b) and 2(c)). In addition, the positive ratio of CKS2 staining (score of 3-6) in HCC tissues (78.2%, 122/156) was significantly higher (*P* < 0.001) than that of corresponding paracancerous tissues (37.2%, 58/156). Consistent with prior analyses, the overexpression of CKS2 was also observed in HCC tissues at the protein level.

### 3.3. CKS2 Was Correlated with Clinicopathological Features in HCC

The correlation of CKS2 with clinicopathological features in 156 HCC patients is demonstrated in Table 1. High expression of CKS2 was significantly associated with liver cirrhosis (*χ*^2^ = 5.695, *P* = 0.019), poor differentiation (*χ*^2^ = 5.436, *P* = 0.020), portal vein invasion (*χ*^2^ = 13.645, *P* < 0.001), advanced TNM stage (*χ*^2^ = 5.531, *P* = 0.019), metastasis (*χ*^2^ = 7.035, *P* = 0.008), and recurrence (*χ*^2^ = 9.112, *P* = 0.003). However, CKS2 expression was not associated with patients' age, gender, AFP, HBV infection, or tumor size.

### 3.4. High CKS2 Expression Associated with Poor Survival in Patients with HCC

**Figure 3:**
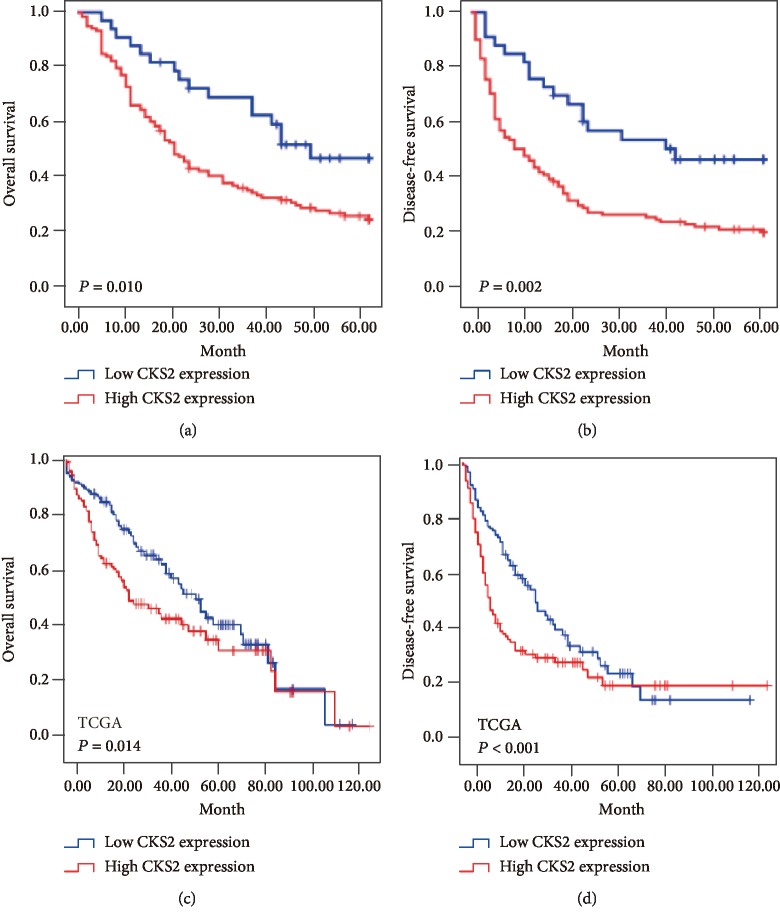
Overall survival and disease-free survival curves for 156 HCC patients. (a) Kaplan-Meier analysis of overall survival (OS) according to high or low CKS2 expression in 156 HCC patients. (b) Kaplan-Meier analysis of disease-free survival (DFS) according to high or low CKS2 expression in 156 HCC patients. (c) Kaplan-Meier curves of OS according to high or low CKS2 expression in the TCGA cohort. (d) Kaplan-Meier curves of DFS according to high or low CKS2 expression in the TCGA cohort. CKS2: cyclin-dependent kinase regulatory subunit 2.

The overall survival (OS) and disease-free survival (DFS) of HCC patients analyzed by using Kaplan-Meier survival curves are elucidated in Figure 3. As shown in Figures 3(a) and 3(b), patients with high CKS2 expression had poorer OS (*P* = 0.01) and DFS (*P* = 0.002) than those cases with low CKS2 expression. Consistently, the survival analyses of TCGA databases also revealed that high CKS2 expression level was significantly correlated with lower OS (*P* = 0.014) and DFS (*P* < 0.001) in HCC patients (Figures 3(c) and 3(d)). These results demonstrated that high expression of CKS2 indicated poor survival of HCC patients.

### 3.5. CKS2 Served as an Independent Prognostic Marker in HCC Patients

Univariant and multivariant Cox regression analyses were performed to evaluate potential prognostic factors of HCC (Tables 2 and 3). As shown in Table 2, univariate analysis indicated that tumor size, differentiation, HBV infection, AFP, portal vein invasion, TNM stage, metastasis, and CKS2 expression were associated with OS of HCC patients. Further multivariate analyses demonstrated that CKS2 expression (*P* = 0.014), tumor size (*P* = 0.021), portal vein invasion (*P* = 0.007), and TNM stage (*P* = 0.01) were independent prognostic factors for overall survival of HCC patients.

Similarly, for DFS of HCC patients, univariate analyses revealed that CKS2 expression, tumor size, AFP, portal vein invasion, HBsAg, TNM stage, and metastasis were potential factors. Subsequent multivariate Cox analysis indicated that CKS2 (*P* = 0.002), together with portal vein invasion (*P* = 0.038), and TNM stage (*P* < 0.001) were independent prognostic factors for disease-free survival of 156 HCC patients (Table 3). Based on the analyses above, we proposed that CKS2 was an independent prognostic factor for HCC patients.

### 3.6. Potential Roles of CKS2 in HCC Progression

**Figure 4:**
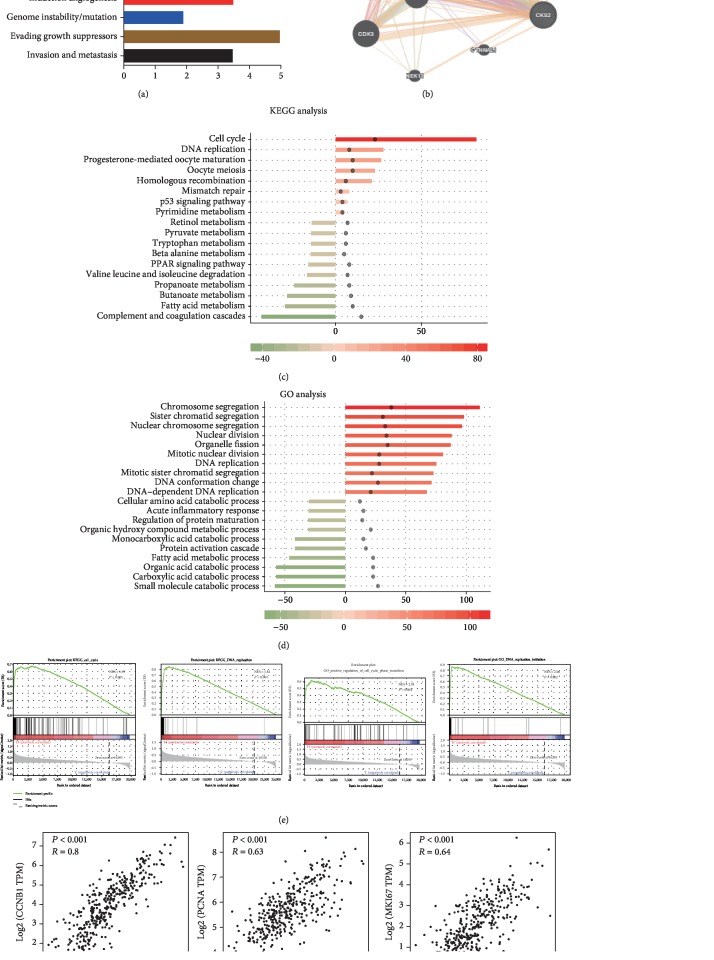
Potential roles of CKS2 in HCC progression. (a) The possible roles of CKS2 in tumors were analyzed by the Cancer Hallmarks Analytics Tool. (b) Then, protein interaction analysis of CKS2 was predicted by the GeneMANIA tool. (c) GO annotations based on the top 116 upregulated and downregulated genes associated with CKS2 expression levels. (d) KEGG pathway analysis based on the top 116 upregulated and downregulated genes that were associated with CKS2 expression levels. (e) Gene set enrichment analysis (GSEA) of TCGA datasets elucidated that CKS2 was implicated in cell cycle and DNA replication pathways. (f) The correlation of CKS2 with proliferative markers CCNB1, PCNA, and Ki-67 was analyzed by the GEPIA tool. CKS2: cyclin-dependent kinase regulatory subunit 2; NES: normalized enrichment score.

We further investigated the possible roles of CKS2 in HCC progression (Figure 4**)**. Cancer Hallmarks Analytics Tool showed that CKS2 might be associated with proliferation, resisting cell death, angiogenesis, invasion, and metastasis (Figure 4(a)). Then, the protein interaction analysis indicated that CKS2 might interact with some cell cyclin-related proteins, including CCNB1, CCNA2, CDK1, and CDK2 (Figure 4(b)). To explore the molecular functions related to CKS2, we identified the differentially expressed genes between CKS2-high and CKS2-low groups. Then, we performed the GO and KEGG analysis based on the top 116 genes that were positively or negatively associated with CKS2. GO enrichment found that the top involved biological processes included the chromosome segregation, DNA replication, catabolic process, and fatty acid metabolic process (Figure 4(c)). Pathway analyses suggested the top significant pathways consisting of the cell cycle, DNA replication, complement and coagulation cascade, and fatty acid metabolism pathways (Figure 4(d)). Furthermore, GSEA elucidated that CKS2 was implicated in cell cycle and DNA replication pathways (Figure 4(e)). As shown in Figure 4(f), CKS2 had significantly positive correlation with proliferative markers CCNB1, PCNA, and Ki-67 in HCC tissues, suggesting that CKS2 might contribute to HCC progression by regulating cell proliferation.

### 3.7. CKS2 Facilitated Malignant Features of HCC Cells

**Figure 5:**
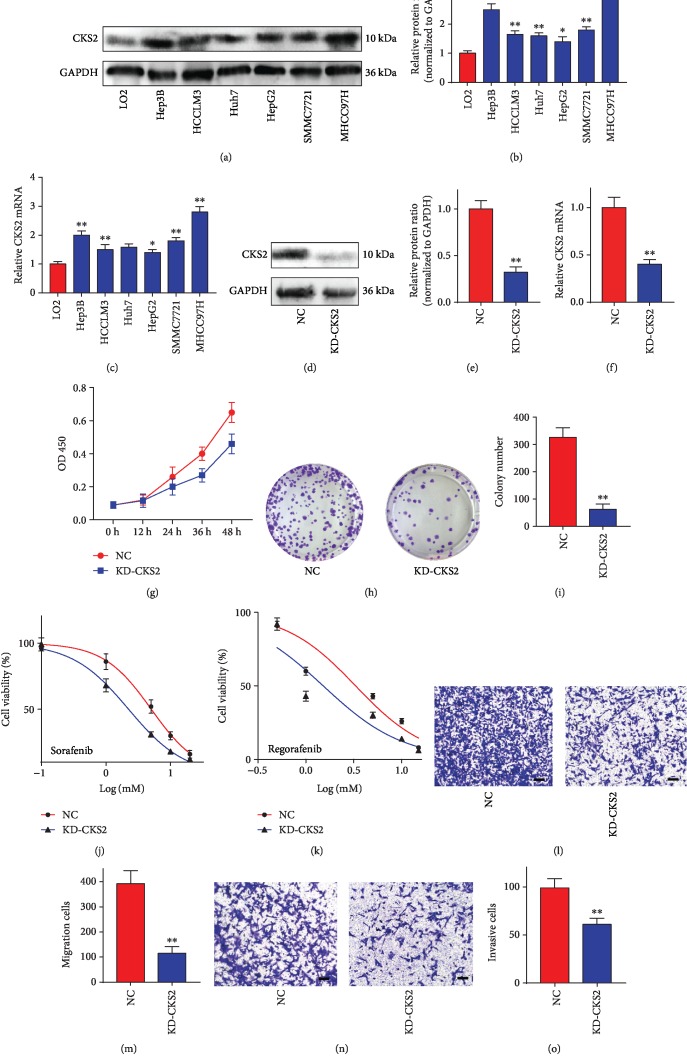
CKS2 promoted malignant behaviors of HCC cells. (a) The CKS2 expression of HCC cell lines was detected by western blotting. (b) The intensity of each bar in (a). (c) The CKS2 mRNA expression in HCC cell lines was detected by RT-qPCR. (d) CKS2 expression of MHCC97H cells transfected with KD-CKS2 plasmid was measured by western blotting. (e) The intensity of each bar in (d). (f) CKS2 expression of MHCC97H cells was detected by RT-qPCR. (g) CCK8 assay was conducted to determine the proliferation of MHCC97H cells transfected with NC and KD-CKS2. (h) The representative images of MHCC97H cell-derived colonies. (i) The colony number derived from cells transfected with NC or KD-CKS2. (j) The viability of MHCC97H cells with sorafenib treatment. (k) The viability of MHCC97H cells with regorafenib treatment. (l) The representative images of migration cells in the Transwell assay. (m) The number of migration cells. (n) The representative images of invasive cells in the Transwell assay. (o) The number of invasive cells. CKS2: cyclin-dependent kinase regulatory subunit 2. ^∗^*P* < 0.05; ^∗∗^*P* < 0.01.

Based on the bioinformatic analysis above, we further validated the effects of CKS2 on HCC cells. As shown in Figures 5(a) and 5(b), CKS2 expression in HCC cell lines was significantly higher than that of the normal liver cell LO2, in which MHCC97H had the highest protein expression of CKS2 (*q* = 17.96, *P* = 0.0001). Consistently, dramatically elevated mRNA levels of CKS2 were also observed in MHCC97H cells (*q* = 15.94, *P* = 0.0001, Figure 5(c)). Then, following transfection with the KD-CKS2 plasmid, CKS2 expression of MHCC97H cells was obviously downregulated at both the mRNA and protein levels (Figure 5(d)–5(f)). The CCK-8 assay demonstrated that the proliferation of MHCC97H cells was significantly inhibited with the knockdown of CKS2 (Figure 5(g)). KD-CKS2 also impaired the colony formation ability of MHCC97H cells (*t* = 11.34, *P* = 0.0003, Figures 5(h) and 5(i)). Additionally, knockdown of CKS2 enhanced the sensitivity of MHCC97H cells against sorafenib (IC50: 5.043 *μ*M vs. 2.042 *μ*M; Figure 5(j)) and regorafenib (IC50: 3.303 *μ*M vs. 1.517 *μ*M; Figure 5(k)). Furthermore, Transwell assays demonstrated that silencing CKS2 remarkably downregulated the migrative (*t* = 8.127, *P* = 0.0012, Figures 5(l) and 5(m)) and invasive (*t* = 5.686, *P* = 0.0047, Figures 5(n) and 5(o)) abilities of MHCC97H cells. These results indicated that CKS2 might promote the malignant phenotypes of HCC cells.

## 4. Discussion

HCC is one of the most common and malignant primary liver tumors with resistance to chemotherapy, especially for the cases at advanced stages. While patients benefit from some targeted drugs, HCC frequently develops tolerance to the currently available drug administration and subsequently leads to a poor prognosis [21]. Thus, it is imperative to find a prognostic marker and even a novel target for HCC.

There is accumulating evidence that CKS2 is elevated in various cancer types, including colorectal, prostate, and bladder cancers [22–24]. However, its expression features and clinical significance in HCC has not been studied thoroughly. According to our analyses in multiple datasets, HCC tissues had higher CKS2 mRNA levels in comparison to normal liver tissues. Interestingly, CKS2 also gradually increased in the order from normal, inflammation, cirrhosis, dysplasia, and early stage HCC to advanced stage, suggesting that CKS2 might contribute to the progression of HCC.

Consistently, we also identified the overexpression of CKS2 protein in 156 HCC cases by the immunochemical staining analysis. Moreover, elevated CKS2 levels were significantly associated with various clinicopathological features, including liver cirrhosis, differentiation, portal vein invasion, TNM stage, and metastasis. CKS2 was previously recommended as a prognostic marker in colorectal, breast, gastric, and esophageal cancers [22, 25–27]. Thus, this study further discovered its roles in predicting the survival of HCC patients. As expected, CKS2 overexpression was obviously correlated with poor OS and DFS in the current HCC cohort. For HCC cases in TCGA cohort, similarly, high expression of CKS2 also led to a poorer outcome in contrast to low CKS2 expression. Taken together, CKS2 might be a prognostic marker to predict survival and recurrence of HCC patients.

CKS2 has been implicated in promoting the aggressive behaviors of cancer cells. Overexpression of CKS2 contributed to the tumor proliferation, migration, and invasion in thyroid cancer [28]. It could also accelerate tumorigenesis of squamous cell carcinoma *in vivo* [26]. While CKS2 was linked to HCC growth by recent studies [19, 29], the possible mechanism remained partially unclear. In our study, we initially explored the underlying roles of CKS2 in HCC. The bioinformatic analyses identified that CKS2 was implicated in proliferation, angiogenesis, invasion, and metastasis. Additionally, enrichment analysis in KEGG pathways and GO terms revealed that CKS2-correlated genes were enriched in the cell cycle and DNA replication pathways, which are known as important factors in the proliferation of tumor cells. Besides, CKS2 was positively correlated with proliferative markers Ki-67 and PCNA, suggesting the potential role of CKS2 in HCC progression.

To validate the bioinformatic analysis, we further explored the roles of CKS2 in HCC cell lines. CKS2 overexpression was observed in HCC cell lines compared with normal hepatocytes. Consistent with the bioinformatic prediction, knockdown of CKS2 obviously downregulated the proliferation, colony formation, and invasion of MHCC97H cells. Moreover, silencing CKS2 also enhanced the efficacy of sorafenib against HCC cells. It was in line with the previous study that CKS2 promoted chemoresistance of cervical cancer [30]. For the mechanisms predicted by bioinformatic methods, CKS2 might interact with various cell cyclin protein family members, subsequently contributing to HCC progression. These evidences indicated that knockdown of CKS2 inhibited the malignant phenotypes of HCC cells.

In conclusion, CKS2 overexpression was significantly correlated with aggressive clinical features and malignant behaviors of HCC cells, suggesting that it might contribute to the progression of HCC. In addition, the current study was the first to evaluate CKS2 as a novel biomarker for OS and DFS of HCC patients. While the results are promising and attractive, further investigations are required to validate its clinical significance in a lager cohort and explore the underlying mechanisms regarding aggressive phenotypes of HCC.

## Figures and Tables

**Table 1:** Clinicopathological features of CKS2 expression in 156 HCC tissues.

Group	*n*	Pos. *n* (%)	*χ* ^2^ value	*P* value
*Age*				
≤50	80	66 (82.50)	1.777	0.183
>50	76	56 (73.68)		
*Gender*				
Female	19	14 (73.68)	0.259	0.611
Male	137	108 (78.83)		
*AFP (ng/mL)*				
≤20	61	49 (80.33)	0.265	0.607
>20	95	73 (76.84)		
*HBsAg*				
Negative	23	18 (78.26)	0.000	0.994
Positive	133	104 (78.20)		
*Tumor size*				
≤5 cm	83	63 (75.90)	0.551	0.458
>5 cm	73	59 (80.82)		
*Liver cirrhosis*				
Without	38	35 (92.11)	5.695	**0.019**
With	118	87 (73.73)		
*Differentiation degree*				
Well	44	29 (65.91)	5.436	**0.020**
Moderate & poor	112	93 (83.04)		
*Portal vein invasion*				
Without	61	57 (93.44)	13.645	**<0.001**
With	95	65 (68.42)		
*TNM*				
I & II	102	74 (72.55)	5.531	**0.019**
III & IV	54	48 (88.89)		
*Metastasis*				
Without	127	94 (74.02)	7.035	**0.008**
With	29	28 (96.55)		
*Recurrence*				
No	79	54 (68.35)	9.112	**0.003**
Yes	77	68 (88.31)		

CKS2: cyclin-dependent kinase regulatory subunit 2; Pos. *n* (%): positive number (%); TNM: tumor node metastasis; AFP: alpha-fetoprotein. Bold: *P* < 0.05.

**Table 2:** Univariate and multivariate analyses for identifying the risk factors of overall survival in 156 HCC patients.

Group	Univariate			Multivariate (gender and sex adjusted)		
	HR	*P*	95% CI	HR	*P*	95% CI
*Gender*						
Male vs. female	1.315	0.411	0.685-2.524			
*Age (years)*						
≤50 vs. >50	0.754	0.155	0.510-1.113			
*Tumor diameter (cm)*						
≤5 vs. >5	3.060	**<0.001**	2.038-4.595	1.706	**0.021**	1.085-2.684
*Differentiation*						
Well vs. moderate & poor	1.886	**0.008**	1.184-3.005	1.567	0.068	0.968-2.538
*AFP (ng/mL)*						
≤50 vs. >50	1.799	**0.005**	1.191-2.716	1.448	0.095	0.937-2.235
*Liver cirrhosis*						
Yes vs. no	1.010	0.963	0.654-1.561			
*Portal vein invasion*						
Yes vs. no	2.068	**0.001**	1.357-3.151	1.919	**0.007**	1.196-3.078
*HBsAg*						
Yes vs. no	2.340	**0.011**	1.217-4.499	1.279	0.494	0.632-2.587
*TNM*						
I-II vs. III-IV	4.468	**<0.001**	2.950-6.766	2.393	**0.001**	1.445-3.963
*Metastasis*						
Yes vs. no	3.016	**<0.001**	1.917-4.745	1.304	0.327	0.767-2.219
*CKS2 expression*						
High vs. low	5.668	**<0.001**	2.943-10.916	2.088	**0.014**	1.161-3.754

CKS2: cyclin-dependent kinase regulatory subunit 2; TNM: tumor node metastasis; AFP: alpha-fetoprotein. Bold: *P* < 0.05.

**Table 3:** Univariate and multivariate analyses for identifying the risk factors of disease-free survival in 156 HCC patients.

Group	Univariate			Multivariate (gender and sex adjusted)		
	HR	*P*	95% CI	HR	*P*	95% CI
*Gender*						
Male vs. female	1.230	0.498	0.675-2.240			
*Age (years)*						
≤50 vs. >50	0.746	0.125	0.513-1.085			
*Tumor diameter (cm)*						
≤5 vs. >5	2.622	**<0.001**	1.782-3.860	1.434	0.131	0.898-2.288
*Differentiation*						
Well vs. moderate & poor	1.486	0.074	0.962-2.295			
*AFP (ng/mL)*						
≤50 vs. >50	1.660	**0.012**	1.120-2.460	1.403	0.108	0.929-2.119
*Liver cirrhosis*						
Yes vs. no	0.903	0.642	0.588-1.387			
*Portal vein invasion*						
Yes vs. no	1.565	**0.025**	1.057-2.315	1.614	**0.038**	1.028-2.536
*HBsAg*						
Yes vs. no	2.088	**0.021**	1.118-3.898	1.216	0.574	0.615-2.403
*TNM*						
I-II vs. III-IV	4.030	**<0.001**	2.697-6.022	2.702	**<0.001**	1.608-4.539
*Metastasis*						
Yes vs. no	2.370	**<0.001**	1.531-3.668	0.846	0.534	0.499-1.434
*CKS2 expression*						
High vs. low	2.210	**0.003**	1.316-3.711	2.511	**0.002**	1.414-4.458

CKS2: cyclin-dependent kinase regulatory subunit 2; TNM: tumor node metastasis; AFP: alpha-fetoprotein. Bold: *P* < 0.05.

## Data Availability

The data used to support the findings of this study are available from the corresponding author upon request.
